# Supplementary material for: Loss of Endothelial TDP-43 Leads to Blood Brain Barrier Defects in Mouse Models of Amyotrophic Lateral Sclerosis and Frontotemporal Dementia
Source: bioRxiv. 2023 Dec 14:2023.12.13.571184. Preprint. [Version 1] doi: 10.1101/2023.12.13.571184 (PMC10760101; doi:10.1101/2023.12.13.571184)
Supplement: Supplement 1 [file NIHPP2023.12.13.571184v1-supplement-1.pdf]

bioRxiv preprint doi: https://doi.org/10.1101/2023.12.13.571184; this version posted December 14, 2023. The copyright holder for this preprint (which was not certified by peer review) is the author/funder, who has granted bioRxiv a license to display the preprint in perpetuity. It is made available under aCC-BY 4.0 International license.

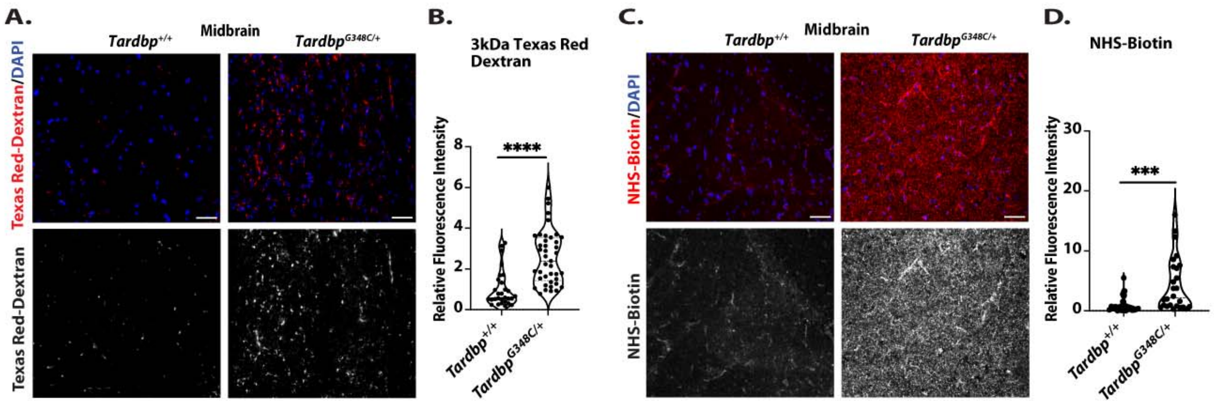

SI Figure 1. Assessment of Blood-Brain Barrier Permeability. (A) The representative immunofluorescence images of 3kDa Texas Red-dextran leakage in mouse midbrain sections from 10-11-month-old mice (n=3 *Tardbp*$^{+/+}$ and n=3 *Tardbp*$^{G348C/+)}$ are shown. (C) NHS-Biotin. (B, D) Quantification of data, with each data point representing the fluorescence image intensity in one image. multiple images per mouse. Scale bars, 50 µm. Data are presented as means ± SEM. Statistical analysis was conducted using an unpaired Mann Whitney test, with significance levels indicated as follows: ***P 0.0001, ****P<0.0001.

bioRxiv preprint doi: https://doi.org/10.1101/2023.12.13.571184; this version posted December 14, 2023. The copyright holder for this preprint (which was not certified by peer review) is the author/funder, who has granted bioRxiv a license to display the preprint in perpetuity. It is made available under aCC-BY 4.0 International license.

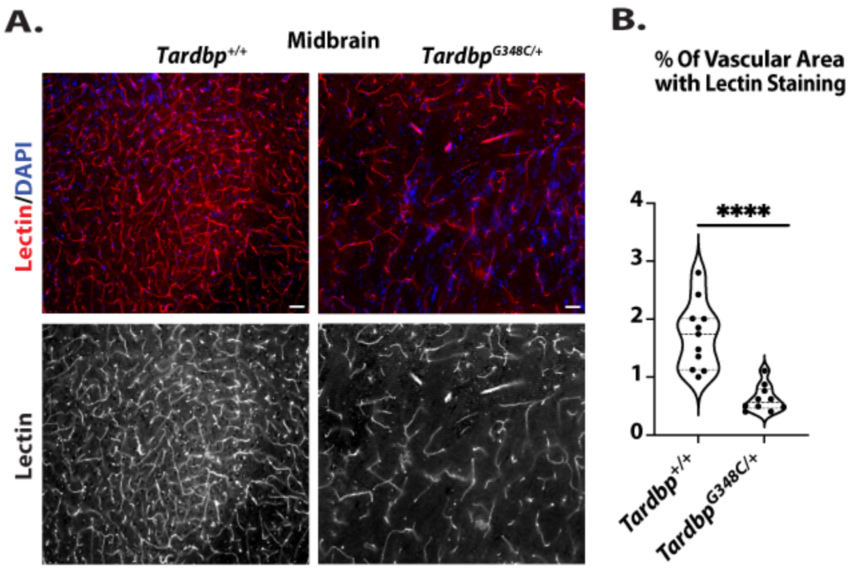

SI Figure 2. Evaluation of Tomato-Lectin Perfusion. (A) The representative immunofluorescence images of Tomato-lectin perfusion in mouse midbrain sections from 10-11-month-old mice (n=3 *Tardbp*$^{+/+}$ and n=3 *Tardbp*$^{G348C/+)}$ are shown. (B) Quantification of data, with each data point representing the fluorescence image intensity in one image. Multiple images per mouse. Scale bars, 50 µm. Data are presented as means ± SEM. Statistical analysis was conducted using an unpaired Mann Whitney test, with significance levels indicated as follows: ****P<0.0001.

bioRxiv preprint doi: https://doi.org/10.1101/2023.12.13.571184; this version posted December 14, 2023. The copyright holder for this preprint (which was not certified by peer review) is the author/funder, who has granted bioRxiv a license to display the preprint in perpetuity. It is made available under aCC-BY 4.0 International license.

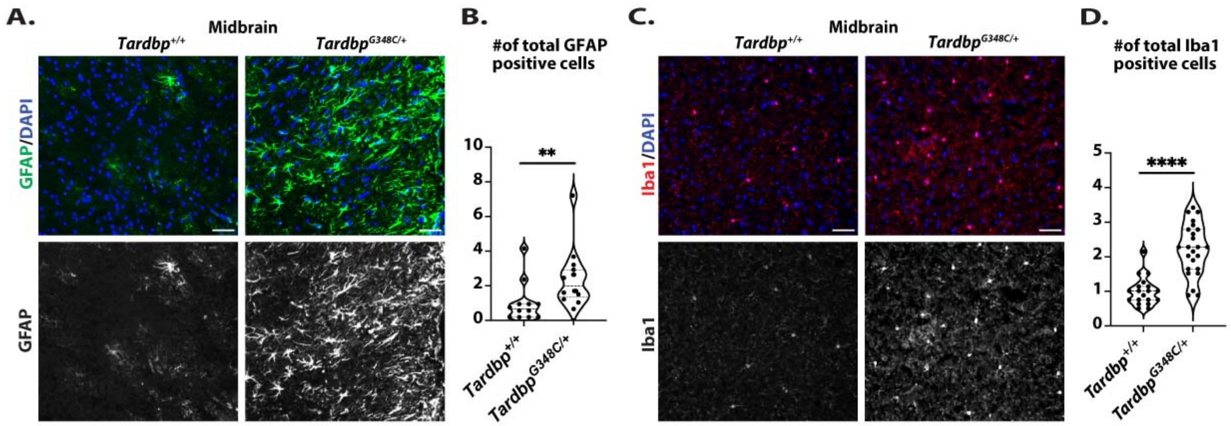

SI Figure 3. Assessment of Astrocyte (GFAP) and Microglia (Iba1) Activation. (A) The representative immunofluorescence images of GFAP staining of astrocytes, and (C) Iba1 staining of microglia in the mouse midbrain reveal consistent results across *Tardbp*$^{+/+}$ mice (n=3) and *Tardbp*$^{G348C/+}$ mice (n=3). (B, D) Quantification of data with each data point representing the number of activated cells in an image. multiple images per mouse. Scale bars, 50 µm. Data are presented as means ± SEM. Statistical analysis was conducted using an unpaired Mann Whitney test, with significance levels indicated as follows: **P 0.0023, ****P<0.0001.

bioRxiv preprint doi: https://doi.org/10.1101/2023.12.13.571184; this version posted December 14, 2023. The copyright holder for this preprint (which was not certified by peer review) is the author/funder, who has granted bioRxiv a license to display the preprint in perpetuity. It is made available under aCC-BY 4.0 International license.

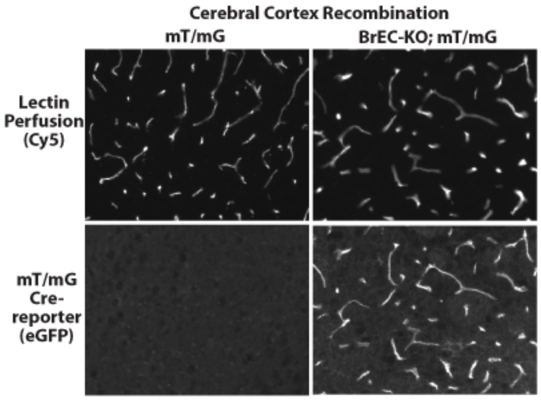

SI Figure 4. Slco1c1-CreERT2 is active in cortical vasculature. (A) BrEC-KO mice with mT/mG Cre reporter were treated with Tamoxifen and collected with Dylight649 lectin perfusion 12 weeks later. eGFP indicates Cre activity in cortical vessels.

bioRxiv preprint doi: https://doi.org/10.1101/2023.12.13.571184; this version posted December 14, 2023. The copyright holder for this preprint (which was not certified by peer review) is the author/funder, who has granted bioRxiv a license to display the preprint in perpetuity. It is made available under aCC-BY 4.0 International license.

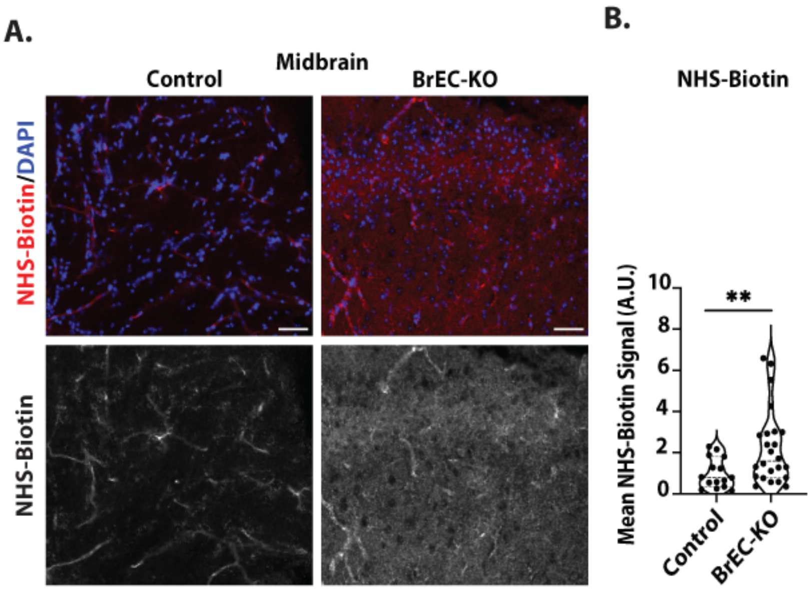

SI Figure 5. Blood brain barrier disruption in *Tardbp* BrEC-KO, midbrain. (A) The representative immunofluorescence images of NHS-biotin leakage in mouse midbrain sections from 3-7-month-old mice (n=3 BrEC-KO and n=3 littermate controls) are shown. B) Quantification signal, with each data point representing the fluorescence image intensity from one image, multiple images per mouse. Scale bars, 50 µm . Data is presented as means ± SEM. Statistical analysis was performed using an unpaired two-tailed Mann Whitney test, with significance levels indicated as follows: **P?0.0096.

bioRxiv preprint doi: https://doi.org/10.1101/2023.12.13.571184; this version posted December 14, 2023. The copyright holder for this preprint (which was not certified by peer review) is the author/funder, who has granted bioRxiv a license to display the preprint in perpetuity. It is made available under aCC-BY 4.0 International license.

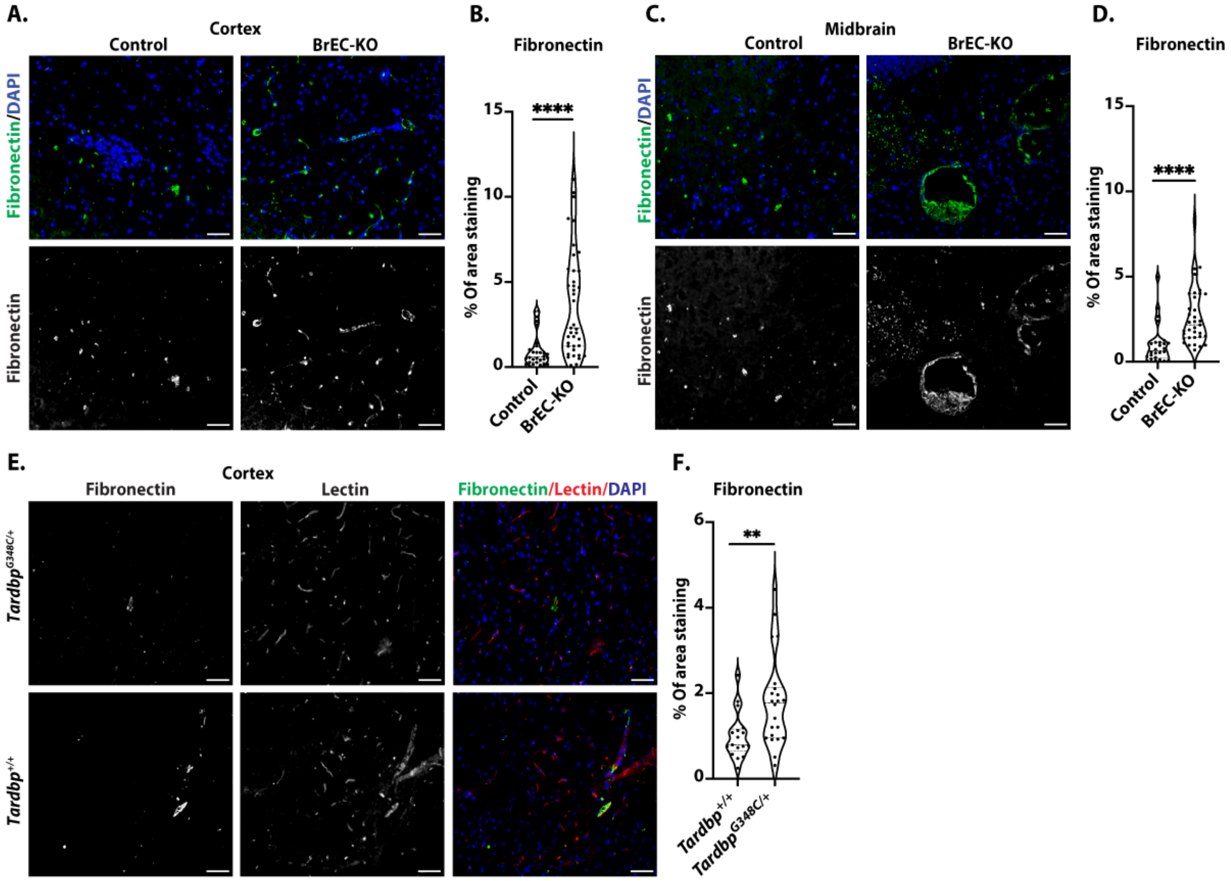

SI Figure 6. Increased Fibronectin Expression in *Tardbp* BrEC-KO and *Tardbp*$^{G348C/+}$ mice. (A) Representative immunofluorescence images of fibronectin in mouse cortex and (C) midbrain sections from 8-11-month-old mice (n=3 BrEC-KO and n=3 littermate controls) are presented. Additionally, (E) representative immunofluorescence images of fibronectin in mouse cortex sections from 10-11-month-old mice (n=3 *Tardbp*$^{+/+}$ and n=3 *Tardbp*$^{G348C/+)}$ are shown. (B, D, F) Quantification signal, with each data point representing the fluorescence image intensity from one image, multiple images per mouse. Scale bars, 50 µm. Data is presented as means ± SEM. Statistical analysis was performed using an unpaired two-tailed Mann Whitney test, with significance levels indicated as follows: **P[illegible]0.0044, ****P<0.0001.

bioRxiv preprint doi: https://doi.org/10.1101/2023.12.13.571184; this version posted December 14, 2023. The copyright holder for this preprint (which was not certified by peer review) is the author/funder, who has granted bioRxiv a license to display the preprint in perpetuity. It is made available under aCC-BY 4.0 International license.

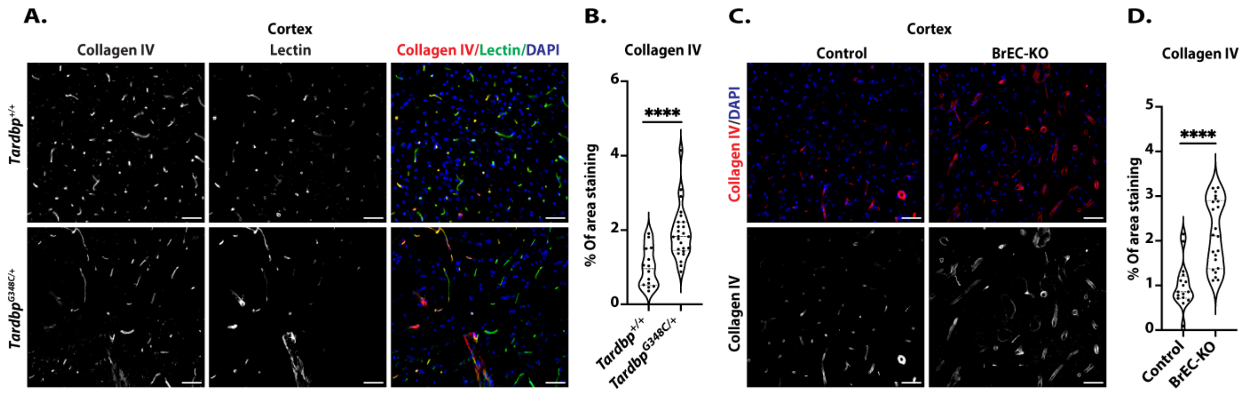

SI Figure 7. Increased Collagen IV Expression in *Tardbp* BrEC-KO and *Tardbp*$^{G348C/+}$ mice. (A) Representative immunofluorescence images of Collagen IV in mouse cortex sections from 10-11-month-old mice (n=3 *Tardbp*$^{+/+}$ and n=3 *Tardbp*$^{G348C/+}$) are presented. Additionally, (C) representative immunofluorescence images of Collagen IV in mouse cortex sections from 8-11-month-old mice (n=3 BrEC-KO and n=3 littermate controls) are shown. (B&D) Quantification signal, with each data point representing the fluorescence image intensity from one image, multiple images per mouse. Scale bars, 50 µm . Data is presented as means ± SEM. Statistical analysis was performed using an unpaired two-tailed Mann Whitney test, with significance levels indicated as follows: ****P<0.0001.

bioRxiv preprint doi: https://doi.org/10.1101/2023.12.13.571184; this version posted December 14, 2023. The copyright holder for this preprint (which was not certified by peer review) is the author/funder, who has granted bioRxiv a license to display the preprint in perpetuity. It is made available under aCC-BY 4.0 International license.

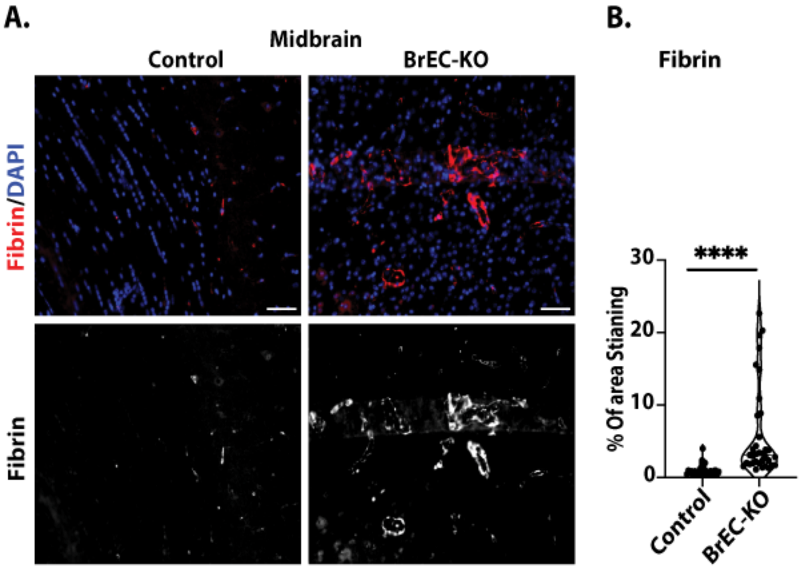

SI Figure 8. Fibrin Deposition in BrEC-KO Mouse Midbrain. (A) Representative immunofluorescence images of fibrin deposition in mouse midbrain sections from 8-11-month-old mice (n=3 BrEC-KO and n=3 littermate controls) are shown. (B) Quantification signal, with each data point representing the fluorescence image intensity from one image, multiple images per mouse. Scale bars, 50 µm. Data is presented as means ± SEM. Statistical analysis was performed using an unpaired two-tailed Mann Whitney test, with significance levels indicated as follows: ****P<0.0001.

bioRxiv preprint doi: https://doi.org/10.1101/2023.12.13.571184; this version posted December 14, 2023. The copyright holder for this preprint (which was not certified by peer review) is the author/funder, who has granted bioRxiv a license to display the preprint in perpetuity. It is made available under aCC-BY 4.0 International license.

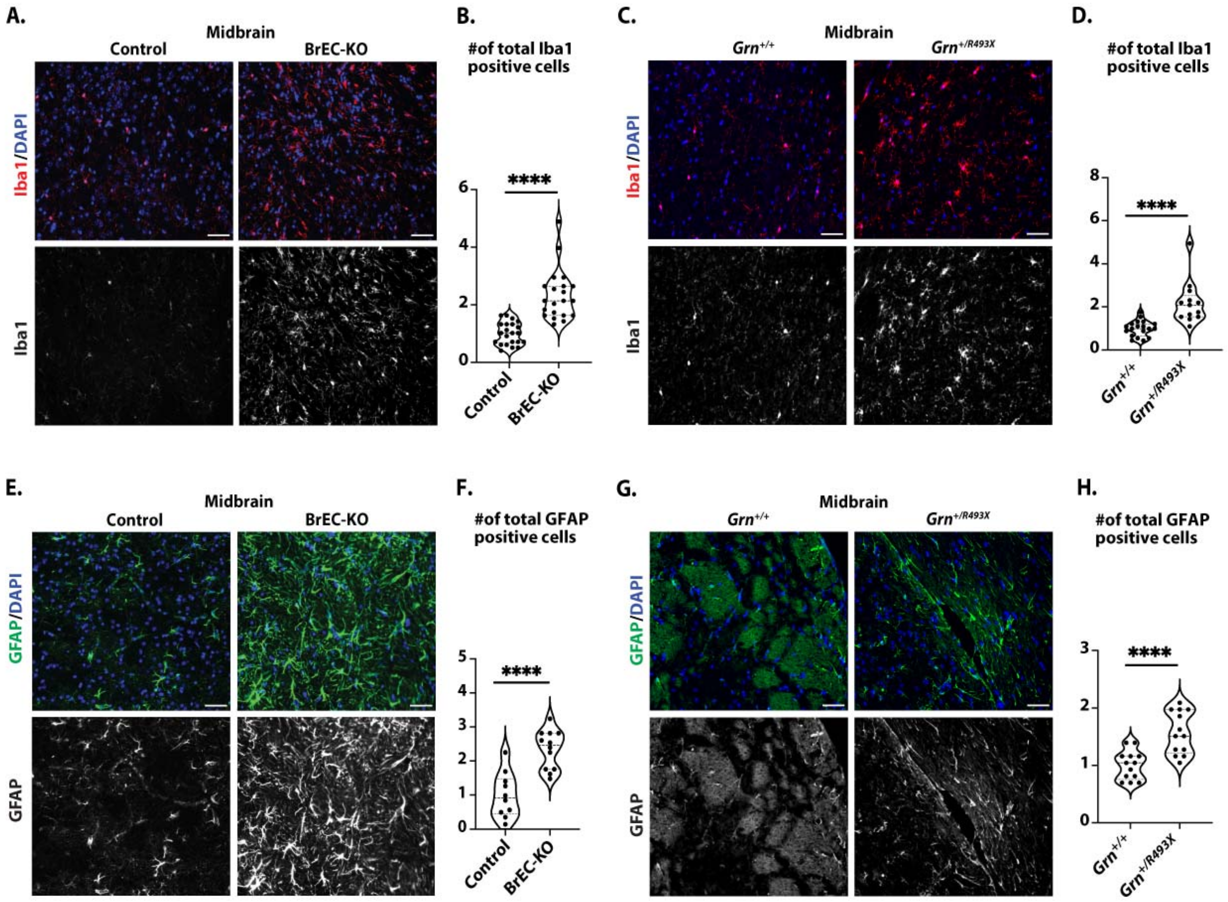

SI Figure 9. Assessment of Astrocyte (GFAP) and Microglia (Iba1) Activation. (A, C) Representative immunofluorescence images of Iba1 staining of microglia in the mouse midbrain reveal consistent results across (n=3 BrEC-KO and littermate controls mice (n=3), as well as n=3 *Grn*$^{R493X/+}$ and littermate controls mice (n=3) and (E, G) GFAP staining of astrocytes reveals a substantial increase in astrocyte numbers, resembling astrogliosis observed in FTD. (B, D, F, H) Quantification of data with each data point representing the number of activated cells in an image. multiple images per mouse. Scale bars, 50 µm. Data are presented as means ± SEM. Statistical analysis was conducted using an unpaired two-tailed Mann Whitney test, with significance levels indicated as follows: ****P<0.0001.

bioRxiv preprint doi: https://doi.org/10.1101/2023.12.13.571184; this version posted December 14, 2023. The copyright holder for this preprint (which was not certified by peer review) is the author/funder, who has granted bioRxiv a license to display the preprint in perpetuity. It is made available under aCC-BY 4.0 International license.

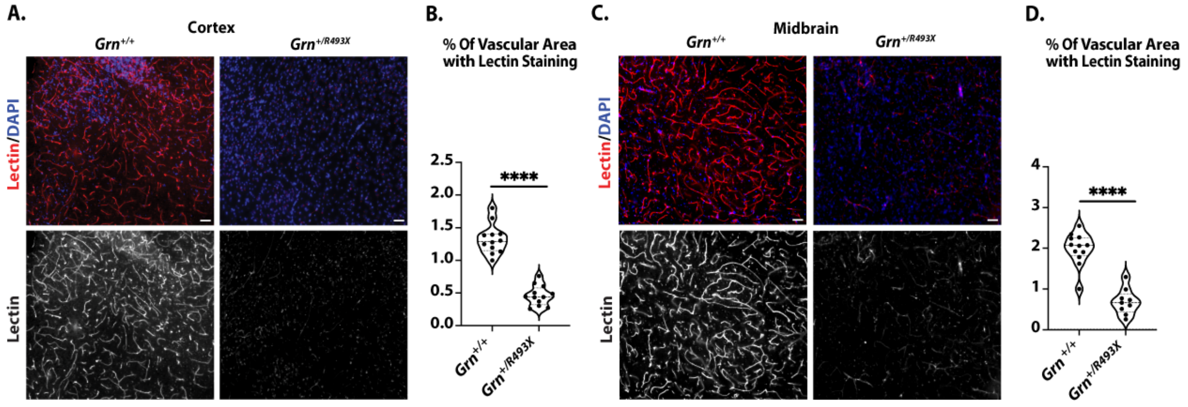

SI Figure 10. Evaluation of Tomato-Lectin Perfusion. (A) The representative immunofluorescence images of Tomato-lectin perfusion in mouse midbrain sections from 25–27-month old mice (n=3 *Grn*$^{R493X/+}$ and littermate control mice (n=3) are shown. (B) Quantification of data, with each data point representing the fluorescence image intensity in one image. Multiple images per mouse. Scale bars, 50 µm. Data are presented as means ± SEM. Statistical analysis was conducted using an unpaired Mann Whitney test, with significance levels indicated as follows: ****P<0.0001

bioRxiv preprint doi: https://doi.org/10.1101/2023.12.13.571184; this version posted December 14, 2023. The copyright holder for this preprint (which was not certified by peer review) is the author/funder, who has granted bioRxiv a license to display the preprint in perpetuity. It is made available under aCC-BY 4.0 International license.

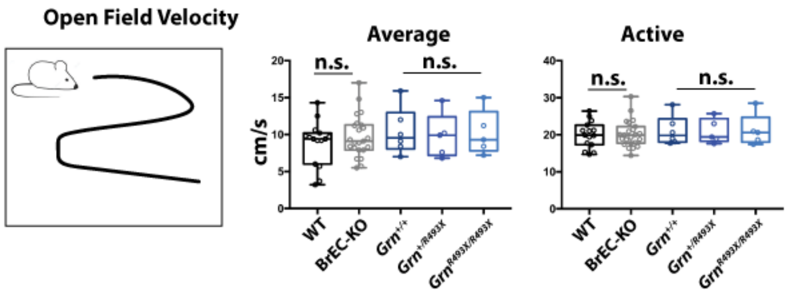

SI Figure 11. Behavioral Assessment of BrEC-KO Mice, Littermate Controls, and *Grn*+/R493X Knock-in Mouse Model of Frontotemporal Lobe Dementia (FTLD) in Open Field Velocity. BrEC-KO and littermate controls were assessed in behavioral core along with a knock-in mouse model of Frontal Temporal Lobe Dementia (FTLD, *Grn*+/R493X). No difference was observed in open field velocity.
